# Differentiation between high- and low-grade urothelial carcinomas using contrast enhanced ultrasound

**DOI:** 10.18632/oncotarget.20151

**Published:** 2017-08-10

**Authors:** Qiuyang Li, Jie Tang, Enhui He, Yanmi Li, Yun Zhou, Baojun Wang

**Affiliations:** ^1^ Department of Ultrasound, Chinese People’s Liberation Army General Hospital, Beijing, China; ^2^ Department of Ultrasound, Beijing Friendship Hospital, Capital Medical University, Beijing, China; ^3^ Department of Urology, Chinese People’s Liberation Army General Hospital, Beijing, China

**Keywords:** contrast-enhanced ultrasonography, urothelial carcinoma, enhancement pattern, time-intensity curve

## Abstract

**Purpose:**

To evaluate the value of contrast-enhanced ultrasonography (CEUS) in the differentiation of high and low grade urothelial carcinoma.

**Materials and Methods:**

192 with 192 bladder lesions, including 110 high grade urothelial carcinoma and 82 low grade urothelial carcinoma were examined by CEUS. Among 192 tumors, enhancement patterns of 96 tumors between August 2010 and December 2012 were analyzed retrospectively. Then from January 2013 to April 2015, compared with CEUS was performed on 96 tumors for prospective differential diagnosis. Sensitivity, specificity, accuracy, positive predictive value and negative predictive value were assessed.

**Results:**

With the CEUS view, dominant enhancement patterns were revealed as fast wash-in and slow wash-out for high grade urothelial carcinoma, fast wash-in and fast wash-out for low grade urothelial carcinoma, respectively. At CEUS, the prospective differentiation of bladder tumors showed sensitivity 86% , specificity 90%, accuracy 88%, positive predictive value 92%, and negative predictive value 82% for high grade tumors, while sensitivity 85% , specificity 89%, accuracy 88%, positive predictive value 85% and negative predictive value 89% for low grade tumors, respectively.

**Conclusions:**

Our study demonstrates the great potential of CEUS in the differentiation of high and low grade urothelial carcinoma. Since CEUS is an effective, inexpensive, and non-invasive method. It could be a reliable tool in the evaluation of patients with bladder tumors.

## INTRODUCTION

Bladder tumors are the most common malignancies of the urinary system, with age-standardized rates (ASR) of 23.6 in men and 5.4 in women in western countries [1]. Bladder tumors may be epithelial or mesenchymal, and over 95% are of the epithelial type [2]. Patients with non-muscle invasive bladder cancer (NMIBC) are usually treated with endoscopic resection and surveillance, whereas patients with muscle invasive bladder cancer (MIBC) often undergo radical extirpative surgery. Generally, 20% of high-grade tumors will progress to MIBC during treatment or follow-up. Thus, accurate preoperative staging and grading is important for optimizing treatment strategies for these tumors [3–5].

Cystoscopy is currently the most sensitive method for detecting bladder tumors, and transurethral resection of bladder tumors (TURB) remains a reliable method for establishing tumor stage and grade. However, TURB is invasive and requires sedation or anesthesia. CT, MRI, and conventional ultrasound (US) have also been used to assess bladder tumors, but these methods are primarily used for staging and not classification [6–8]. However, CT and MRI, which are performed using intravascular contrast agents, can accurately detect bladder tumor neovascularization suggestive of progression [9, 10]. US is rarely used for this purpose, because its specificity is low for distinguishing between benign and malignant bladder tumors [8]. More recently developed contrast-enhanced ultrasound (CEUS), which can visualize blood flow status in minor blood vessels, is an effective method for classifying focal versus diffuse lesions [11–13]. However, there are few guidelines regarding the use of contrast agents in CEUS for bladder tumor classification, and the value of CEUS in the differential diagnosis of low- versus high-grade bladder carcinomas has not been clarified. To assess the potential role of CEUS in characterizing bladder tumors, we retrospectively established diagnostic criteria based on dominant enhancement patterns. We subsequently adopted these criteria for the differentiation of bladder tumors in a prospective study.

## RESULTS

Sonographic examinations were well tolerated by all patients, and no side effects related to the contrast agent were observed.

### Retrospective study

**Figure 1:**
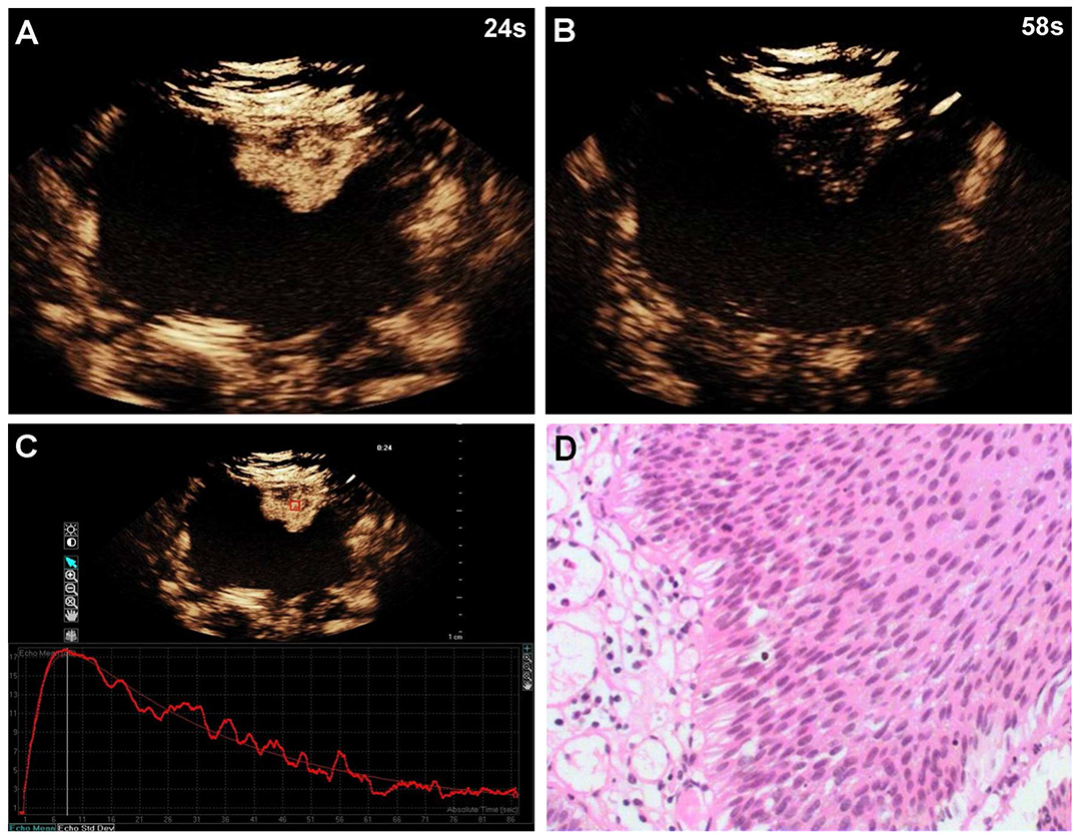
A 60-year-old woman with low-grade bladder cancer on the anterior bladder wall CEUS shows strong and fast lesion enhancement during the late arterial phase (24 sec) **(A)**, and complete wash-out during the venous phase (58 sec) **(B)**. Time-intensity curve shows fast wash-in and fast wash-out, and lower PI **(C)**. Surgical specimen confirming low-grade bladder cancer (hematoxylin & eosin staining, original magnification ×100) **(D).**

**Figure 2:**
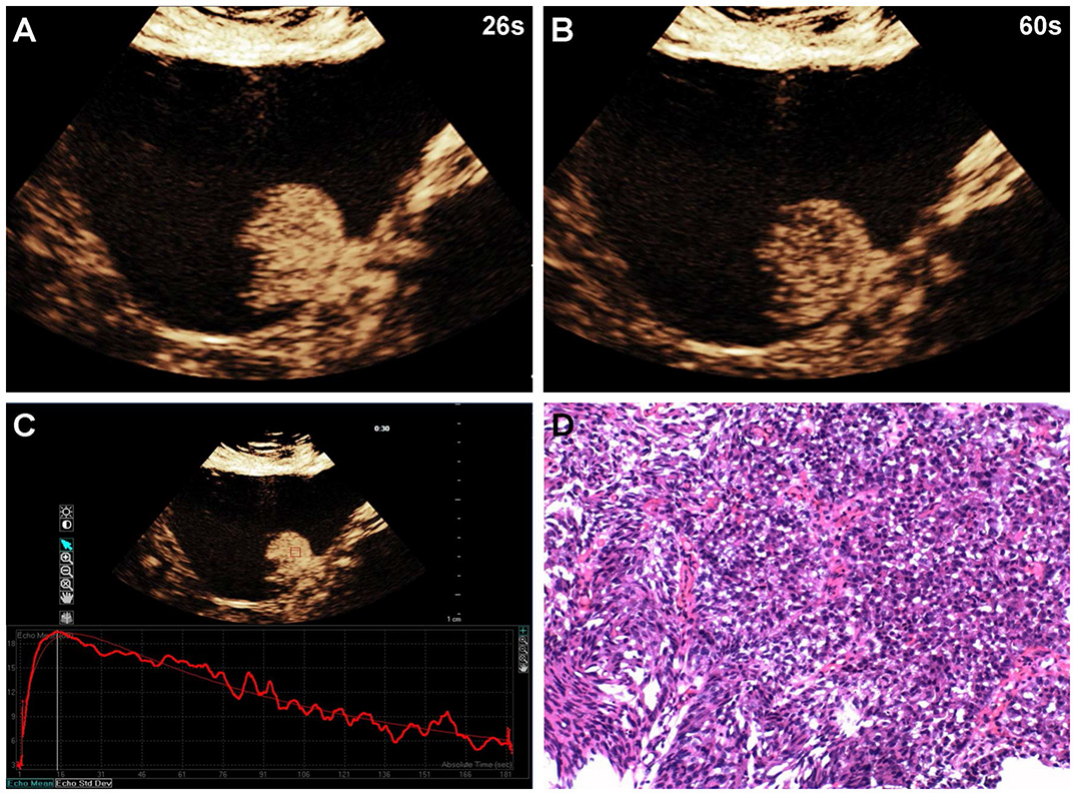
A 58-year-old man with high-grade bladder cancer on the posterior bladder wall CEUS shows strong and homogeneous lesion enhancement during the late arterial phase (26 sec) **(A)** and slow wash-out during the venous phase (60 sec) **(B)**. Time-intensity curve shows fast wash-in and slow wash-out, and higher PI **(C)**. Surgical specimen confirming high-grade bladder cancer (hematoxylin & eosin staining, original magnification ×100) **(D).**

Of the 96 histopathological lesions, 54 were cases of high-grade urothelial carcinoma (4 Ta, 44 T1, 6 T2) and 42 were low-grade urothelial carcinomas (40 Ta, 2 T1) (Table 1). CEUS patterns were divided into three types: type I, fast wash-in and fast wash-out, 86% (36/42) of low-grade urothelial carcinomas showed quick enhancement in and around the lesions, and regression occurred earlier than in adjacent normal bladder wall (Figure 1); type II, slow wash-in and fast wash-out, 7% (4/54) of high-grade tumors and 5% (2/42) of low-grade tumors showed slow enhancement, and regression occurred earlier than in adjacent normal bladder wall; and type III, fast wash-in and slow wash-out, 85% (46/54) of high-grade urothelial carcinomas were quickly enhanced from the central tumor to its periphery, and regression occurred later than in adjacent normal bladder wall (Figure 2). Comparisons of all parameters of the gamma variate are shown in Table 2. There were no differences in AT and TTP between high- and low-grade bladder tumors (*P*>0.05). High-grade tumor PIs were greater compared to low-grade tumors, and low-grade tumor WTs were less than those of high-grade tumors (*P*<0.05). Combined enhancement patterns for high- versus low-grade bladder tumors are shown in Table 1 .

**Table 1:** Clinical characteristics of patients and bladder lesions in retrospective and prospective studies (mean ± SD )

	Retrospective study ( n = 96 )		Prospective study ( n = 96 )	
	HG	LG	HG	LG
No. of patients	54	42	56	40
No. of lesions	54	42	56	40
No. of papillary lesions	28	20	28	20
No. of sessile lesions	26	22	28	20
Age of patients (yr)	68.7±5.6	63.8±12.3	69.5±8.3	63.2±14.1
Lesion diameter (mm)	23.7±11.7	15.4±8.0	22.2±8.5	14.8±7.8
Final diagnosis				
Surgery	40	4	42	4
Biopsy	14	38	16	36

HG: low grade urothelial carcinoma; LG: low grade urothelial carcinoma.

**Table 2:** Enhancement patterns using CEUS in two phases: predictive value in retrospective study

Enhancement patterns		Positive predictive value	
Arterial phase	Venous phase	HG	LG
Fast wash-in	Fast wash-out	0.10 (4 )	0.90 (36)
Slow wash-in	Fast wash-out	0.67 (4)	0.33 (2)
Fast wash-in	Slow wash-out	0.92 (46)	0.08 (4)

HG: low grade urothelial carcinoma; LG: low grade urothelial carcinoma.

### Prospective study

Of the 96 lesions, 56 were high-grade urothelial carcinomas (6 Ta, 45 T1, 5 T2) and 40 were low-grade urothelial carcinomas (38 Ta, 2 T1). CEUS, based on fast wash-in and slow wash-out criteria for distinguishing high-grade urothelial carcinomas, yielded a sensitivity of 86%, specificity of 90%, accuracy of 88%, positive predictive value of 92%, and negative predictive value of 82%. Fast wash-in and fast wash-out criteria for detecting low-grade urothelial carcinomas resulted in a sensitivity of 85%, specificity of 89%, accuracy of 88%, positive predictive value of 85%, and negative predictive value of 89% (Table 3).

**Table 3:** Contrast parameters of high and low grade papillary urothelial carcinoma (mean ± SD )

TIC parameters	HG	LG	P value
AT (s)	22.13 ± 1.76	21.44 ± 1.34	0.385
PI (dB)	19.85 ± 12.13	13.87 ± 9.42	0.006
TTP(s)	29.31 ± 2.14	27.06 ± 2.18	0.297
WT(s)	34.83 ± 10.41	20.18 ± 8.27	0.002

AT: arrival time; TTP: time to peak; PI: peak intensity; WT: washout time.

## DISCUSSION

The present study explored CEUS, a newly developed imaging modality, for characterizing different types of bladder lesions.TURB is currently the only reliable method for establishing bladder tumor stages and grades. Conservative treatment is used for non-muscle invasive lesions (Ta and T1), while cystectomy is applied for muscle invasive lesions (T2 and higher stages). Stage T1 tumors may be high grade in 50% of patients at the time of diagnosis, with a 30–50% progression rate [14]. Accurate tumor grading is therefore extremely important for determining individual patient treatment strategies.

Angiogenesis is essential for tumor growth, and neovascularization can occur in both benign and malignant tumors. Color Doppler ultrasound has been used to evaluate patients with bladder lesions due to its low cost and high patient compliance rates. However, color Doppler ultrasound is reportedly not effective in clinical evaluation of bladder tumors [15, 16]. Drudi, *et al.* [17] found that color Doppler ultrasound achieved good sensitivity, but poor specificity for differentiating low- from high-grade bladder tumors. With the development of contrast agents, enhanced CT, dynamic MRI, and CEUS offer new options for evaluating tumor vascularity [9, 10, 18, 19]. While CT and MRI are more commonly used to determine extravesical extension and tumor staging, CEUS can also be used to assess tumor neovascularization. Compared to CT and MRI, CEUS offers improved availability, speed, and real time analysis options, does not require radiation, and can be used to evaluate patients with renal failure. US contrast agents also show high sensitivities for detecting tumor microvascularization [20], and are used routinely to evaluate microvascularization in bladder cancers and other urologic malignancies [21]. In 2008, the European Federation of Societies for Ultrasound in Medicine and Biology (EFSUMB) [22] updated their guidelines to include recommendations for the use of CEUS in bladder lesion differential diagnosis and staging.

Thus far, only two studies have reported on the use of CEUS in bladder cancer detection [17, 23]. Drudi, *et al.* concluded that CEUS reliably differentiates between low- and high-grade bladder carcinomas, and displays typical enhancement patterns. In the present study, we retrospectively characterized bladder lesion enhancement patterns using CEUS, and used the dominant enhancement patterns as diagnostic criteria for our prospective study. CEUS for bladder lesion characterization in the prospective study yielded a good sensitivity, specificity, accuracy, positive predictive value, and negative predictive value, favoring the application of CEUS in differentiating bladder lesions. In the prospective study, 48/56 high-grade tumor cases showed the dominant fast wash-in and slow wash-out enhancement pattern and 34/40 low-grade cases showed the dominant fast wash-in and fast wash-out enhancement pattern. Contrast enhancement quantitation in ROIs via QLAB software appeared reliable. Although both low- and high-grade bladder lesions are hypervascular, some characteristic features, including WT and PI, are helpful in differentiating these tumors. Since contrast enhancement lasted longer in high-grade bladder tumors, high-grade bladder tumor WT was typically longer than that of low-grade bladder tumors. PI in the time-intensity curve reflected total contrast agent entering the lesion. In high-grade bladder tumors, vascularity developed quickly, with small lumens, thin walls, an incomplete endothelium, and without smooth muscle cells or nerve terminals. These vascularity features result in systolic and diastolic dysfunction, which may increase blood perfusion in the arterial phase. Low-grade tumors were associated with low flow rates and straight- and regularly-organized vessels. Total contrast agent amounts and PIs were lower in low- than in high-grade tumors.

This study had limitations. First, we failed to delineate tumor stages, although precise staging of bladder tumors is very important for treatment planning and outcome prediction. We differentiated high- versus low-grade bladder tumors using CEUS [24]. Second, in cases that presented with multiple tumors, we chose the largest tumor for use in our study. However, NMIBCs are typically larger tumors and invasive high-grade tumors tend to be smaller, so choosing the largest tumors in these cases may have skewed our sample set. Third, our CEUS diagnostic value assessment focused only on high- and low-grade bladder tumors, which can exhibit similar imaging findings and are easily misdiagnosed. Differential diagnoses from other kinds of bladder tumors, especially those with complex appearances, such as urothelial papillomas and papillary urothelial neoplasms of low-malignancy, should also be assessed.

In summary, our results indicate that CEUS using the contrast agent SonoVue might offer a clinically useful method for characterizing tumor vascularity and differentiating between high- and low-grade bladder tumors. Confirmation of our findings will require further investigation with larger patient cohorts.

## MATERIALS AND METHODS

### Subjects

This study was approved by the Institutional Review Board of the PLA General Hospital in Beijing, China. Informed consent was obtained from all patients included in the study. Between August 2010 and December 2012, 104 consecutive patients who were known or suspected to have bladder tumors were examined via CEUS. Of these, 96 patients (78 men and 18 women) with bladder tumors were retrospectively enrolled based on the following criteria: satisfactory CEUS images were acquired without artifacts; the diagnosis of bladder tumors was confirmed according to the reference standard; and the tumors had not been treated previously. Eight patients were excluded (four bladder tumors without a histological diagnosis, two benign tumors suspicious of urothelial papilloma, and two tumors suspicious of papillary utothelial neoplasm of low malignancy). For 16 patients with multiple tumors, only the largest tumor with CEUS images and definitive diagnosis was selected for evaluation. The dominant enhancement patterns in CEUS were summarized for the 96 tumors, and were used as diagnostic criteria in the prospective study.

From January 2013 to April 2015, a CEUS prospective study was conducted on 104 consecutive patients with suspicious bladder tumors detected by prior conventional US or CT. For 20 patients with multiple lesions, CEUS was performed on the largest tumor. Exclusion criteria were as follows: patients without appropriate CEUS images due to artifacts; patients with lesions requiring histopathological diagnosis, but surgery or biopsy was not possible due to poor heart function or absence of consent. Finally, 96 patients (80 men and 16 women) with bladder lesions were enrolled. Four patients were excluded due to absence of consent, two due to poor heart function for surgery, and two with urothelial papilloma. Final diagnoses and clinical characteristics are shown in Table 1.

### Imaging

In both the retrospective and prospective studies, US and CEUS were performed by the same sonographer with five years experience with abdominal CEUS. Patients underwent examinations after ≥200mL fluid ingestion so that bladder filling was sufficient for good visualization of the bladder tumor. No other preparation was necessary. Examinations were performed transabdominally with the patient in supine position. Both US and CEUS were performed using a Philips IU22 system with a 1.0–6.0 MHz probe (Philips Royal Electronic Corporation, Holland). Pulse inversion (PI) and power modulation (PM) modes at a mechanical index of 0.05 were adopted for contrast-specific sonography using the contrast agent, SonoVue (Bracco, Milan, Italy). SonoVue is a second-generation sulfur hexafluoride microbubble contrast agent that provides strong and continuous signal enhancement and allows continuous real-time imaging. A 1.2 mL contrast agent bolus was injected through a 20-gauge cannula followed by 5 mL normal saline flush using a three-way stopcock to ensure that no residual contrast agent was left in the intravenous catheter. Images and cine clips of the entire CEUS examination were stored digitally for offline analysis.

### Image and data analysis

All patients underwent US, which revealed bladder lesion sizes, boundaries, modalities, echo features, and color Doppler flow imaging (CDFI) information. Morphologically, lesions with broader bases compared to their heights were categorized as sessile masses, while lesions with heights greater than their base widths were categorized as papillary masses (Table 4). After CEUS, two off-line readers observed and recorded bladder tumor enhancement and wash-out patterns. Enhancement was compared with that of adjacent normal bladder wall tissue. Both readers were skilled in urological sonography with more than five years of CEUS examination experience, and were blinded to patient final diagnoses and clinical and radiological information. If there was disagreement between the two readers, another pair of senior physicians re-evaluated the clips until a final conclusion was reached. Observation indexes included contrast agent arrival time, time to peak, and wash-out time. The CEUS vascular phases used for staging liver lesions were not applicable for bladder tumors due to blood supply and hemodynamics differences between the liver and bladder. Therefore, in our study, tumor staging was based on both MR findings and our clinical experience. Perfusion patterns were divided into two vascular phases: (1) the arterial phase was defined as maximal hyperechogenicity within the aorta, and began 17–20 sec after contrast agent injection; and (2) the venous phase was defined as the time at which the vascular tree became hypoechoic, and began approximately 30 sec after contrast agent injection. Quantitative analyses of contrast enhancement were carried out in a region of interest (ROI) using the QLAB quantification software (Philips Medical System, Bothell, WA, USA). ROIs in bladder tumors showed hyperperfusion averaging 25.91 mm^2^ in size. Arrival times (AT), peak intensities (PI), times to peak intensity (TTP) and wash-out times (WT) were extracted by the off-line software. AT was defined as the first point of the curve clearly above the baseline intensity, followed by a further rise; PI was defined as maximum intensity; TTP was the time from the start of the injection to the maximum intensity of the curve; and WT was the time from maximum intensity to baseline intensity, which was directly estimated from the time intensity curve.

**Table 4:** Sensitivity, specificity, accuracy, PPV and NPV for differential diagnosis on CEUS in prospective study

Bladder tumors	Diagnostic criteria based on enhancement patterns	Sensitivity	Specificity	Accuracy	PPV	NPV
HG	Fast wash-in and slow wash-out	86% (48/56)	90% (36/40)	88% (84/96)	92% (48/52)	82% (36/44)
LG	Fast wash-in and fast wash-out	85% (34/40)	89% (50/56)	88% (84/96)	85% (34/40 )	89% (50/56)

PPV: positive predictive value; NPV: negative predictive value.

In the retrospective study, enhancement patterns were categorized into two phases and enhancement changes were observed. Arterial phase enhancement patterns consisted of tumor enhancement and TTP, while in the venous phase, the hypoechoic pattern was defined as the lesion having low echogenicity compared with the surrounding bladder wall. After pattern classification, enhancement patterns from the two phases were combined and summarized, and the positive predictive value (PPV) was calculated. The combined enhancement patterns with higher PPVs served as diagnostic criteria for the prospective study. In the prospective study, two readers assessed CEUS images and differentiated bladder lesions according to the diagnostic criteria established in the retrospective study.

### Statistical analysis

Data were exported into Microsoft Excel where means and standard deviations (SD) were calculated. SPSS software package version 11.0 for Windows was used for data analysis. Measurement data were analyzed using *t* test. *P*<0.05 was considered statistically significant.
